# H3K4me2/3 modulate the stability of RNA polymerase II pausing

**DOI:** 10.1038/s41422-023-00794-3

**Published:** 2023-03-15

**Authors:** Shibin Hu, Aixia Song, Linna Peng, Nan Tang, Zhibin Qiao, Zhenning Wang, Fei Lan, Fei Xavier Chen

**Affiliations:** Fudan University Shanghai Cancer Center, Shanghai Key Laboratory of Medical Epigenetics, Human Phenome Institute, Shanghai Key Laboratory of Radiation Oncology, Institutes of Biomedical Sciences, Fudan University, Shanghai, China

**Keywords:** Histone post-translational modifications, Transcription, Epigenetics

Dear Editor,

Modifications of histones are intricately linked with the regulation of gene expression, having demonstrated roles in various physiological processes and pathogenesis. Methylation of histone H3 lysine 4 (H3K4) implemented by the COMPASS family is enriched at promoters and associated cis-regulatory elements, with H3K4 trimethylation (H3K4me3) being a hallmark of active gene promoters.^1^ However, the relative contributions of the deposition and removal of H3K4 methylation to transcriptional control remain unclear. We found that rapid depletion of core COMPASS subunits led to a dynamic turnover of H3K4me2 and H3K4me3 that is mediated by KDM5 demethylases. Depleting H3K4me2 and H3K4me3 did not affect TFIID recruitment or initiating RNA polymerase II (Pol II) but instead reduced levels of paused Pol II, which could be attributed to increased enrichment of the Integrator-PP2A complex (INTAC).

The COMPASS family is composed of SET1A, SET1B, and MLL1–4 methyltransferase-containing complexes, each of which has both redundant and non-redundant functions.^1^ Among the four shared COMPASS subunits, RBBP5 is essential for complex assembly, nucleosome recognition, and catalytic activation, whereas DPY30 flexibly associates with, and stimulates, H3K4 methylation (Fig. 1a).^2,3^ Therefore, to investigate the dynamics of genome-wide H3K4 methylation, we individually integrated the degradation tag FKBP12^F36V^ (dTAG) into the N-terminus of the endogenous *Rbbp5 and Dpy30* loci in mouse embryonic stem cells (mESCs) to allow rapid disruption of COMPASS (Fig. 1a; Supplementary information, Fig. S1a). Surprisingly, 6 h dTAG treatment in mRBBP5-dTAG cells induced a substantial loss in H3K4me2 and H3K4me3, while H3K4me1 exhibited no noticeable change at the bulk level (Fig. 1b). This contrasts with the notion of H3K4me3 being a relatively long-lived histone modification.^4^ Western blotting revealed that H3K4me3 levels declined more rapidly than H3K4me1 and H3K4me2, with near-complete loss of H3K4me3 achieved after 12 h dTAG treatment (Fig. 1c), while the bulk levels of other examined COMPASS subunits (Fig. 1c), KDM5 family members that catalyze H3K4 demethylation (Supplementary information, Fig. S1b, c), and other histone modifications (Supplementary information, Fig. S1c) exhibited no noticeable changes. Importantly, treatment with the pan-KDM5 inhibitor KDM5-C70 restored H3K4me3 levels, indicating that KDM5 demethylases are mainly responsible for RBBP5 degradation-induced rapid H3K4me3 depletion (Supplementary information, Fig. S1d).

**Fig. 1:**
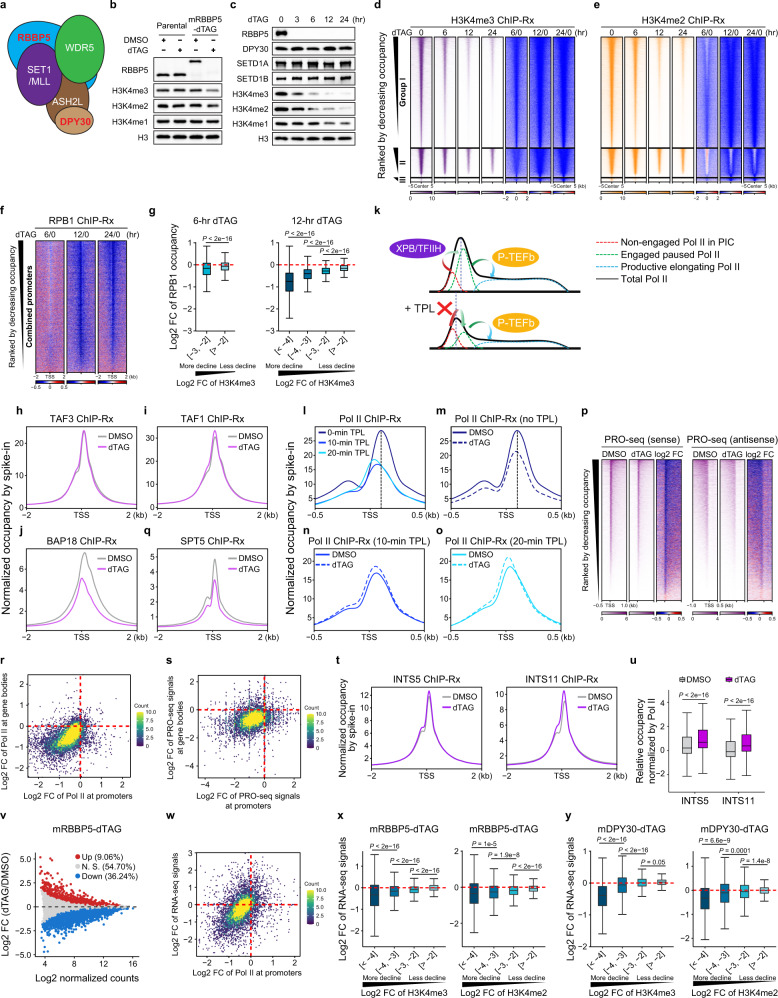
Rapid COMPASS depletion and subsequent loss of H3K4me2/H3K4me3 diminish the occupancy of paused but not initiating Pol II and compromise gene expression. **a** Shared composition of COMPASS family. **b** Western blotting in parental and mRBBP5-dTAG mESCs treated with DMSO or dTAG for 6 h. **c** Western blotting in mRBBP5-dTAG cells with indicated time-course dTAG treatment. **d**, **e** Heatmaps showing the H3K4me3 (**d**) and H3K4me2 (**e**) occupancies (reads per million mapped reads (RPM) per bp and log_2_ fold change (FC)) ranked by decreasing H3K4me3 occupancy in DMSO condition. **f** Log_2_FC heatmaps of Pol II occupancy at combined promoters in mRBBP5-dTAG cells ranked by decreasing Pol II occupancy in DMSO condition. **g** Boxplots showing the correlation of log_2_FC of Pol II and H3K4me3 (grouped based on the extent of decline in H3K4me3 occupancy). **h**–**j**, **q** Metaplots showing the average levels of TAF3 (**h**), TAF1 (**i**), BAP18 (**j**), and SPT5 (**q**) centered at TSS with H3K4me3. **k** Schematic model for the composition of promoter-bound Pol II (top) and the changes upon TPL treatment (bottom). **l**–**o** Average Pol II occupancy upon DMSO/TPL treatment in mRBBP5-dTAG mESCs. **p** Heatmaps of PRO-seq signal (RPM per bp and log_2_FC) for sense and antisense transcription centered at TSS with H3K4me3. **r**, **s** Two-dimensional density plots comparing the log_2_FC of Pol II ChIP-Rx signal (**r**) and PRO-seq signal (**s**) at promoters (x axis) and gene bodies (y axis) for genes with H3K4me3. **t** Average INTS5 and INTS11 occupancies centered at TSS with H3K4me3. **u** Boxplot showing the occupancies of INTS5 and INTS11 compared with Pol II in DMSO/dTAG-treated cells. **v** M (log_2_FC, y axis) versus A (log_2_ normalized counts, x axis) (MA) plot of spike-in normalized RNA-seq showing the changes in gene expression by 24 h dTAG treatment in mRBBP5-dTAG cells. **w** Two-dimensional density plot comparing the log_2_FC of Pol II ChIP-Rx signal at promoters (x axis) and gene expression (y axis) for genes with H3K4me3. **x**, **y** Boxplots showing the correlation of the log_2_FC of RNA-seq signal and H3K4me2/3 (grouped based on the extent of decline in H3K4me2/3 occupancy) in mRBBP5-dTAG (**x**) and mDPY30-dTAG mESCs (**y**).

To monitor H3K4 methylation dynamics genome-wide, we performed ChIP-seq with reference exogenous genome spike-in (ChIP-Rx) in dTAG-treated mRBBP5-dTAG mESCs at different time points. After 6 h of dTAG treatment, all significantly changed peaks of H3K4me3, and most significantly changed peaks of H3K4me2, had decreased occupancy (Supplementary information, Fig. S1e, f). We therefore classified the peaks into three groups using this time point: Group I loci exhibited a decline in both H3K4me2 and H3K4me3, Group II loci only had a reduction in H3K4me3, while Group III loci exhibited no significant changes in H3K4me3 (Supplementary information, Fig. S1g). For Group I peaks, both H3K4me2 and H3K4me3 levels were dramatically reduced at 6 h and reached minimal levels by 12–24 h. Group II peaks had higher average H3K4me2 and H3K4me3 levels in the 0 h condition, and these declined at a slower rate than Group I loci, while H3K4me1 reached a summit at 6 h but gradually decreased thereafter. Group III peaks had very low levels of all three methylation states and were excluded from subsequent analyses (Fig. 1d, e; Supplementary information, Fig. S1h). Consistent with western blotting results, dTAG treatment resulted in rapid depletion of RBBP5 from chromatin for all groups, indicating that group-specific H3K4 methylation dynamics are not due to different RBBP5 degradation efficiencies at specific loci (Supplementary information, Fig. S1i, j).

Acute degradation of a second shared COMPASS subunit, DPY30, also induced a rapid decline in H3K4me3 (Supplementary information, Fig. S2a, b), and KDM5 inhibition restored H3K4me3 levels (Supplementary information, Fig. S2c). Although the overall rate of change in H3K4 methylation states was somewhat slower during dTAG treatment of mDPY30-dTAG compared to mRBBP5-dTAG mESCs, the relative kinetics of mono- vs. di- vs. trimethylation changes are comparable (Supplementary information, Fig. S2d–i). A larger number of peaks showed loss of H3K4me2 and H3K4me3 by 6 h of degradation of RBBP5 (*n* = 13,798) compared to DPY30 (*n* = 11,640), in accordance with the notion of RBBP5 being more central for COMPASS functions (Supplementary information, Fig. S3a).^1,2^ Accordingly, comparisons of H3K4 methylation state changes tested in mRBBP5-dTAG and mDYP30-dTAG cells showed overall positive correlations, but the reductions of H3K4me2 and H3K4me3 induced by RBBP5 depletion were more pronounced (Supplementary information, Fig. S3b–j). We therefore used mRBBP5-dTAG mESCs to further explore biological functions of H3K4 methylation.

We next asked how H3K4 methylation changes impact transcription by performing time-resolved Pol II ChIP-Rx in dTAG-treated mRBBP5-dTAG mESCs. Pol II occupancy at H3K4me3-enriched regions was analyzed separately for promoters and enhancers (Supplementary information, Fig. S4a). Both promoters and enhancers in our previously defined Group I and II loci, exhibited reduced Pol II occupancy by 12 and 24 h dTAG treatment, while 6 h of dTAG treatment had minimal changes (Supplementary information, Fig. S4b–d). Because Pol II changes were equivalently evident for Groups I and II, especially at promoters, we combined the two groups and focused on promoter regions for subsequent analyses, which confirmed the prevalent decrease in Pol II occupancy at promoters after 12 and 24 h of dTAG treatment (Fig. 1f; Supplementary information, Fig. S4e).

Given that changes in H3K4me2 and H3K4me3 levels were first apparent at 6 h of RBBP5 depletion, but the near depletion of these marks in 12–24 h dTAG-treated cells (Supplementary information, Fig. S4f–h) was concomitant with decreased Pol II occupancy, we speculated that a critical level of H3K4 methylation was required to maintain Pol II at promoters. We therefore compared H3K4me2/3 and Pol II changes at promoters after 6 and 12 h of dTAG treatment. Although H3K4 methylation and Pol II occupancy changes correlated at both time points, the greatest decline in Pol II was at 12 h when H3K4 methylation levels had dropped by 16-fold (log_2_ fold change <–4) (Fig. 1g; Supplementary information, Fig. S4i), indicating that the near-complete depletion of H3K4me2/3 is required to disrupt the recruitment and/or maintenance of Pol II at promoters. Supporting the dependency on H3K4me2/3 for the regulation of Pol II occupancy at promoters, the addition of KDM5 inhibitor KDM5-C70 rescued the decline in bulk H3K4me2/3 levels (Supplementary information, Fig. S4j) and Pol II occupancy at promoters (Supplementary information, Fig. S4k) in mRBBP5-dTAG cells with 12 h of dTAG treatment.

H3K4me3 has been proposed to play an instructive role in transcription by facilitating the recruitment of H3K4me3 readers or reader-containing complexes, such as the preinitiation complex (PIC) assembly factor TFIID. Therefore, we sought to determine whether the reduced Pol II occupancy at promoters in COMPASS-disrupted cells can be ascribed to impaired recruitment of TFIID. TAF3, a subunit of TFIID, that directly interacts with H3K4me3,^5,6^ and TAF1, a scaffold subunit of TFIID,^7^ were selected for ChIP-Rx to quantify TFIID occupancy genome wide. BAP18, another reader protein of H3K4me3 was included as a control.^8^ Even after 24 h dTAG-induced RBBP5 degradation, the protein levels and chromatin occupancies of TAF3 or TAF1 exhibited no apparent alterations compared with DMSO-treated cells (Fig. 1h, i; Supplementary information, Fig. S5a–c). Contrastingly, BAP18 levels at promoters declined dramatically (Fig. 1j; Supplementary information, Fig. S5d). We conclude that Pol II loss upon H3K4me2/3 depletion in mESCs is not due to reduced TFIID recruitment.

Pol II ChIP-seq signal reflects a combination of non-engaged polymerases within PIC and transcriptionally engaged but transiently paused Pol II, with the transition from PIC to pausing complex requiring the translocase activity of XPB/TFIIH (Fig. 1k). To determine which type of Pol II is primarily affected in RBBP5-depleted cells, we treated the cells with triptolide (TPL), an inhibitor of the translocase activity of XPB/TFIIH, for 10 and 20 min to prevent new Pol II transitioning to the pausing complex, while preserving Pol II within the PIC. As expected, TPL treatment caused a 5′ shift of Pol II peak centers (Fig. 1l). In stark contrast to the reduced Pol II levels at promoters after RBBP5 degradation without TPL (Fig. 1m), dTAG treatment did not reduce promoter-bound Pol II in the presence of 10 or 20 min TPL-treatment (Fig. 1n, o). These data suggest that H3K4me2/3 loss preferentially impairs the enrichment of paused Pol II rather than the PIC at promoters.

To further assess Pol II in the paused state, we conducted spike-in normalized precision run-on sequencing (PRO-seq) that specifically measures the engaged, paused polymerases but not the PIC form of Pol II. The level of PRO-seq signal decreased profoundly in RBBP5-depleted mESCs in both sense and antisense transcription (Fig. 1p; Supplementary information, Fig. S6a). These changes in PRO-seq signal correlated with overall Pol II levels at promoters, further supporting the notion that the decrease in Pol II at promoters results from loss of the pausing form of Pol II (Supplementary information, Fig. S6b). Accordingly, the level of Pol II phosphorylated at serine 5 of CTD (pSer5), the form of polymerases in the pausing complex, was reduced in dTAG-treated RBBP5-dTAG mESCs (Supplementary information, Fig. S6c).

Paused but not initiating Pol II is tightly associated with SPT5, with SPT5 both stabilizing the paused form of Pol II and subsequently traveling with Pol II after being released into productive elongation.^9,10^ As measured by ChIP-Rx, SPT5 occupancy was diminished upon dTAG-induced RBBP5 degradation and reduction of H3K4me2/3 and Pol II at promoters (Fig. 1q; Supplementary information, Fig. S6d). As illustrated in two-dimensional density plot, the reduced occupancy of SPT5 was overall proportional to that of Pol II at promoters, with most promoters showing reduced levels of both Pol II and SPT5 (Supplementary information, Fig. S6e). These results further support that the loss of H3K4me2 and H3K4me3 specifically affects paused polymerases.

We next sought to scrutinize the fate of paused Pol II loss upon RBBP5 and subsequent H3K4me2/3 depletion. Plotting Pol II occupancy from TSS to TES revealed that RBBP5 degradation led to decreased Pol II levels within gene bodies (Supplementary information, Fig. S7a). Comparison of Pol II ChIP-Rx (Fig. 1r) and PRO-seq (Fig. 1s) data revealed that the decrease in Pol II occupancy at promoters correlated with Pol II changes in gene bodies. Consistently, genes with a greater decline of Pol II at promoters also had greater loss of Pol II in gene bodies (Supplementary information, Fig. S7b, c). These results suggest that the observed loss of paused polymerases in dTAG-treated mRBBP5-dTAG cells is not due to Pol II being released into gene bodies for productive elongation.

The bulk levels of Pol II as assessed by western blotting remained unaltered following RBBP5 depletion, implying that Pol II loss from chromatin is unlikely a result of polymerase protein degradation (Supplementary information, Fig. S7d). Therefore, we surmised that the loss of paused Pol II upon RBBP5 and subsequent H3K4me2/3 loss could be due to premature transcription termination, a pathway undergone by ~80% of paused Pol II.^11^ As the dual-enzymatic INTAC complex is mainly responsible for premature termination at promoters, we measured its occupancy using ChIP-Rx of INTS5, a scaffold subunit of the “Shoulder” module that recruits the phosphatase module, and INTS11, the catalytic subunit of the RNA endonuclease module. In contrast to the reduced levels of paused Pol II and its associating factor SPT5, the binding of INTS5 and INTS11 showed a slight increase following RBBP5 depletion (Fig. 1t; Supplementary information, Fig. S7e, f). Using promoter-bound Pol II and SPT5 for normalization, the relative occupancies of INTS5 and INTS11 exhibited a marked elevation upon RBBP5 depletion compared with Pol II (Fig. 1u) or SPT5 (Supplementary information, Fig. S7g), indicating an increase in the percentage of INTAC-bound Pol II destined for premature termination.^12,13^ These data suggest that the observed decrease in paused Pol II upon H3K4 methylation loss is through premature termination by INTAC.

We next conducted spike-in normalized RNA-seq to examine impact of H3K4 methylation reduction on gene expression. Upon RBBP5 degradation, we observed that the number of significantly downregulated genes was four times as many as significantly upregulated genes (Fig. 1v). Moreover, these changes in expression levels of genes highly correlated with the alterations in Pol II occupancy at promoters (Fig. 1w). Importantly, genes with the most severe decline in H3K4me2/3 levels also exhibited the greatest downregulation of gene expression (Fig. 1x). Consistent with the changes in mRBBP5-dTAG cells, more genes are downregulated than upregulated in dTAG-treated mDPY30-dTAG cells (Supplementary information, Fig. S7h). Furthermore, genes exhibiting a greater loss of H3K4 methylation had a more evident loss of gene expression (Fig. 1y). Together, these results reveal a dependency for H3K4 methylation in the optimal activation of productive transcription through the regulation of paused Pol II.

In summary, using rapid degradation systems, we found that dynamic deposition and removal of histone H3K4 methylation modulates the stability of RNA polymerase II pausing. Paused Pol II dwells at promoters for varying lengths of time, ranging from seconds to minutes, implying that the stability of pausing is subject to dynamic regulation.^11^ Most of the mechanistic studies of pausing have been focused on several Pol II-bound pausing regulatory factors, including but not limited to NELF, DSIF, PAF1C and INTAC.^12,14,15^ Our findings lead us to propose a causal role of H3K4me2/3 in retaining paused Pol II at promoters. Importantly, the loss of the transcriptionally pausing form of Pol II following reductions in H3K4me2/3 levels is accompanied by a further decline in elongating polymerases and the subsequent transcriptional downregulation, suggesting that Pol II in the transcriptionally paused state is terminated prematurely rather than being released into productive transcription elongation. Supporting this notion, the loss of H3K4me2/3 induces a relative enrichment of promoter-bound INTAC complex, which has recently been found to be a major regulator of premature termination in higher organisms.^12^ However, it is still unclear whether H3K4me2/3 regulate pausing directly through modulating chromatin structure or by recruiting effector proteins implicated in paused Pol II stability. In conclusion, the studies presented here identify, while raising new questions about, a previously unrecognized interplay between histone modifying enzymes and the regulation of transcription in metazoans.

## Supplementary information

Supplementary information

## Data Availability

Primary sequencing data and BigWig files are deposited at the GEO depository: GSE222848.
