# Supplementary material for: H3K4me2/3 modulate the stability of RNA polymerase II pausing
Source: Cell Res. 2023 Mar 15;33(5):403–6. doi: 10.1038/s41422-023-00794-3 (PMC10156655; doi:10.1038/s41422-023-00794-3)
Supplement: Supplementary file 1 — Supplementary information [file 41422_2023_794_MOESM1_ESM.pdf]

Supplementary Information

H3K4me2/3 modulate the stability of RNA polymerase II pausing

Shibin Hu[1,#,*], Aixia Song[1,#], Linna Peng[1,#], Nan Tang[1], Zhibin Qiao[1], Zhenning Wang[1], Fei Lan[1], Fei Xavier Chen[1,*]

[1]Fudan University Shanghai Cancer Center, Shanghai Key Laboratory of Medical Epigenetics, Human Phenome Institute, Shanghai Key Laboratory of Radiation Oncology, Institutes of Biomedical Sciences, Fudan University, Shanghai, China

[#]These authors contributed equally: Shibin Hu, Aixia Song, Linna Peng

[*]Correspondence: feixchen@fudan.edu.cn, shibinhu@fudan.edu.cn

Table of Contents:

Figures and figure legends

· Supplementary information, Fig. S1: Acute RBBP5 degradation compromises COMPASS and destabilizes H3K4 methylation

· Supplementary information, Fig. S2: Acute DPY30 degradation compromises COMPASS and destabilizes H3K4 methylation

· Supplementary information, Fig. S3: The reduction of H3K4me2 and H3K4me3 induced by RBBP5 depletion is more pronounced than by DPY30 depletion

· Supplementary information, Fig. S4: RBBP5 depletion and subsequent loss of H3K4me2 and H3K4me3 reduce Pol II levels at promoters

· Supplementary information, Fig. S5: TFIID recruitment is not perturbed upon disruption of COMPASS and the subsequent loss of H3K4me2 and H3K4me3

· Supplementary information, Fig. S6: RBBP5 depletion and subsequent loss of H3K4me2 and H3K4me3 diminish the occupancy of paused Pol II

· Supplementary information, Fig. S7: The loss of H3K4me2 and H3K4me3 compromise gene expression

Materials and Methods

· Culture of mESCs

· Antibodies and reagents

· Genome editing for endogenous knock-in dTAG cells

· Chromatin immunoprecipitation sequencing (ChIP-seq) with reference exogenous genome (ChIP-Rx)

· Transient transcriptome sequencing (TT-seq)

· Precision run-on sequencing (PRO-seq)

· Quantification and statistical analysis

References

Supplementary information, Fig. S1

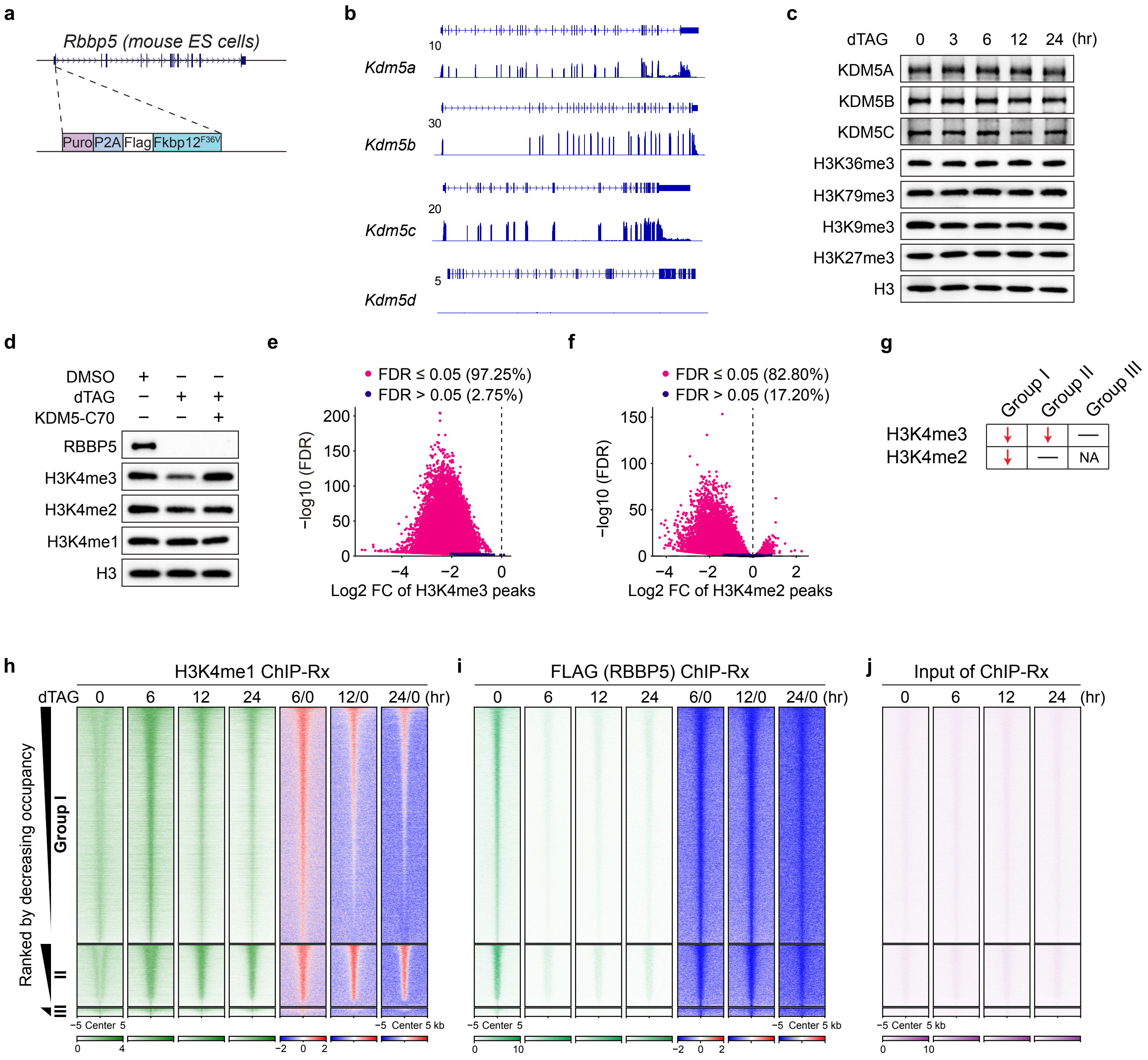

Supplementary information, Fig. S1: Acute RBBP5 degradation compromises COMPASS and destabilizes H3K4 methylation

a Schematic of the generation of knock-in mRBBP5-dTAG mESCs. **b** Genome browser track examples showing the expression levels of *Kdm5a*, *Kdm5b*, *Kdm5c*, and *Kdm5d* by RNA-seq data in mRBBP5-dTAG mESCs. **c** Western blotting of mRBBP5-dTAG mESCs with indicated time-course of dTAG treatment, H3 is a loading control. **d** Western blotting of dTAG treatment with or without pan-KDM5 inhibition by KDM5-C70 in mRBBP5-dTAG mESCs. **e, f** Volcano plots showing the $\log_2$ fold change of H3K4me3 peaks (**e**) and H3K4me2 peaks (**f**) of 6-hour dTAG versus DMSO treatment. **g** Schematic showing the change in patterns of three groups of peaks for 6-hour dTAG versus DMSO treatment. **h, i** Heatmaps showing three groups of H3K4me1 (**h**) and FLAG (RBBP5) (**i**) occupancy (RPM per bp and $\log_2$ fold change) ranked by decreasing H3K4me3 occupancy in the DMSO condition. The peaks are centered at H3K4me3 peak centers. **j** Heatmaps showing three groups of input occupancy (RPM per bp) ranked by decreasing H3K4me3 occupancy in the DMSO condition.

Supplementary information, Fig. S2

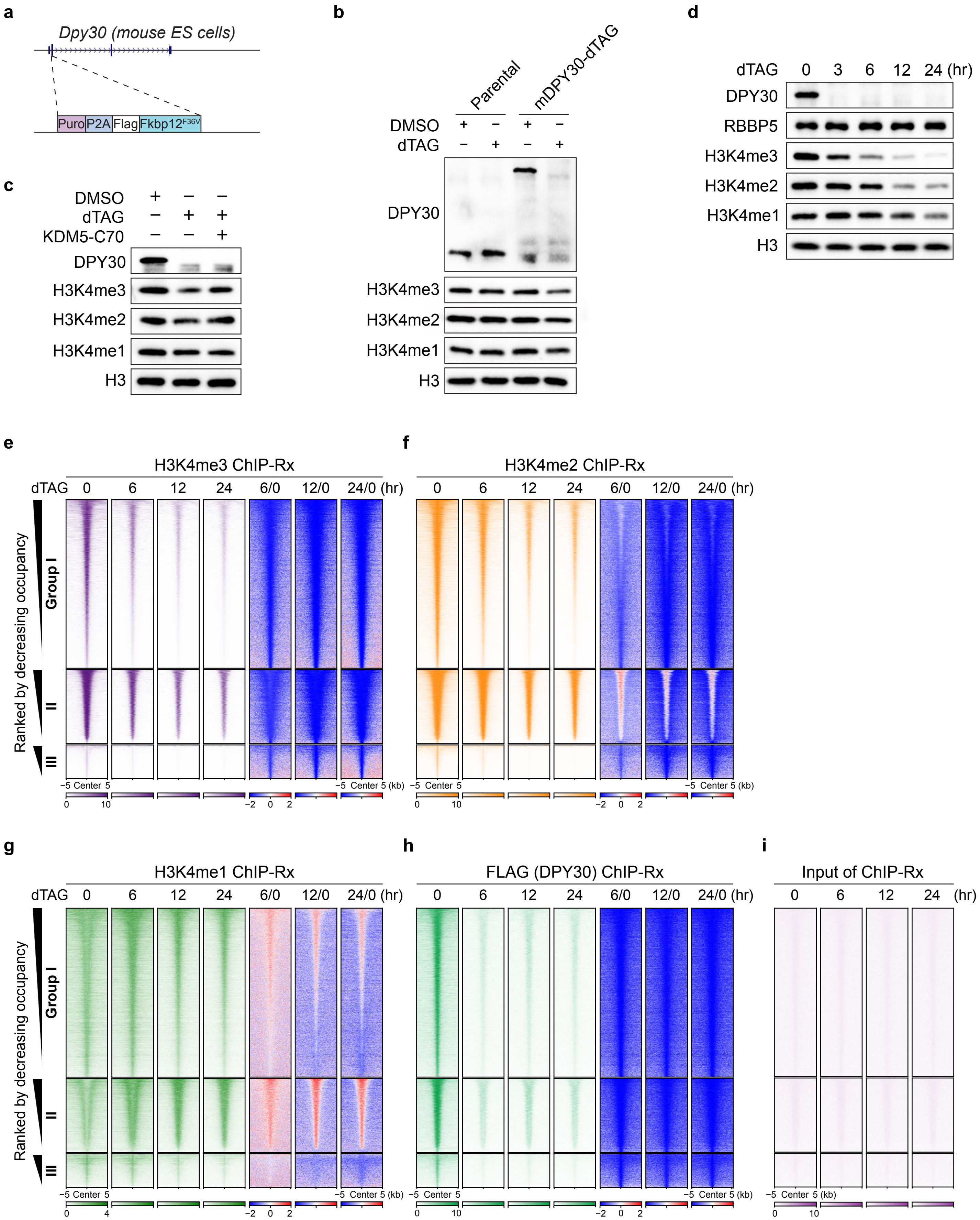

Supplementary information, Fig. S2: Acute DPY30 degradation compromises COMPASS and destabilizes H3K4 methylation

a Schematic of the generation of knock-in mDPY30-dTAG mESCs. **b** Western blotting of whole-cell extracts of parental and mDPY30-dTAG mESCs treated with DMSO or dTAG for 6 hours. H3 is a loading control. **c** Western blotting of dTAG treatment with or without pan-KDM5 inhibition by KDM5-C70 in mDPY30-dTAG mESCs. **d** Western blotting of mDPY30-dTAG mESCs with indicated time-course of dTAG treatment. **e-h** Heatmaps showing three groups of H3K4me3 (**e**), H3K4me2 (**f**), H3K4me1 (**g**), and FLAG (DPY30) (**h**) occupancy (RPM per bp and $\log_2$ fold change) ranked by decreasing H3K4me3 occupancy in the DMSO condition. The peaks are centered at H3K4me3 peak centers. **i** Heatmaps showing three groups of input occupancy (RPM per bp) ranked by decreasing H3K4me3 occupancy in the DMSO condition.

Supplementary information, Fig. S3

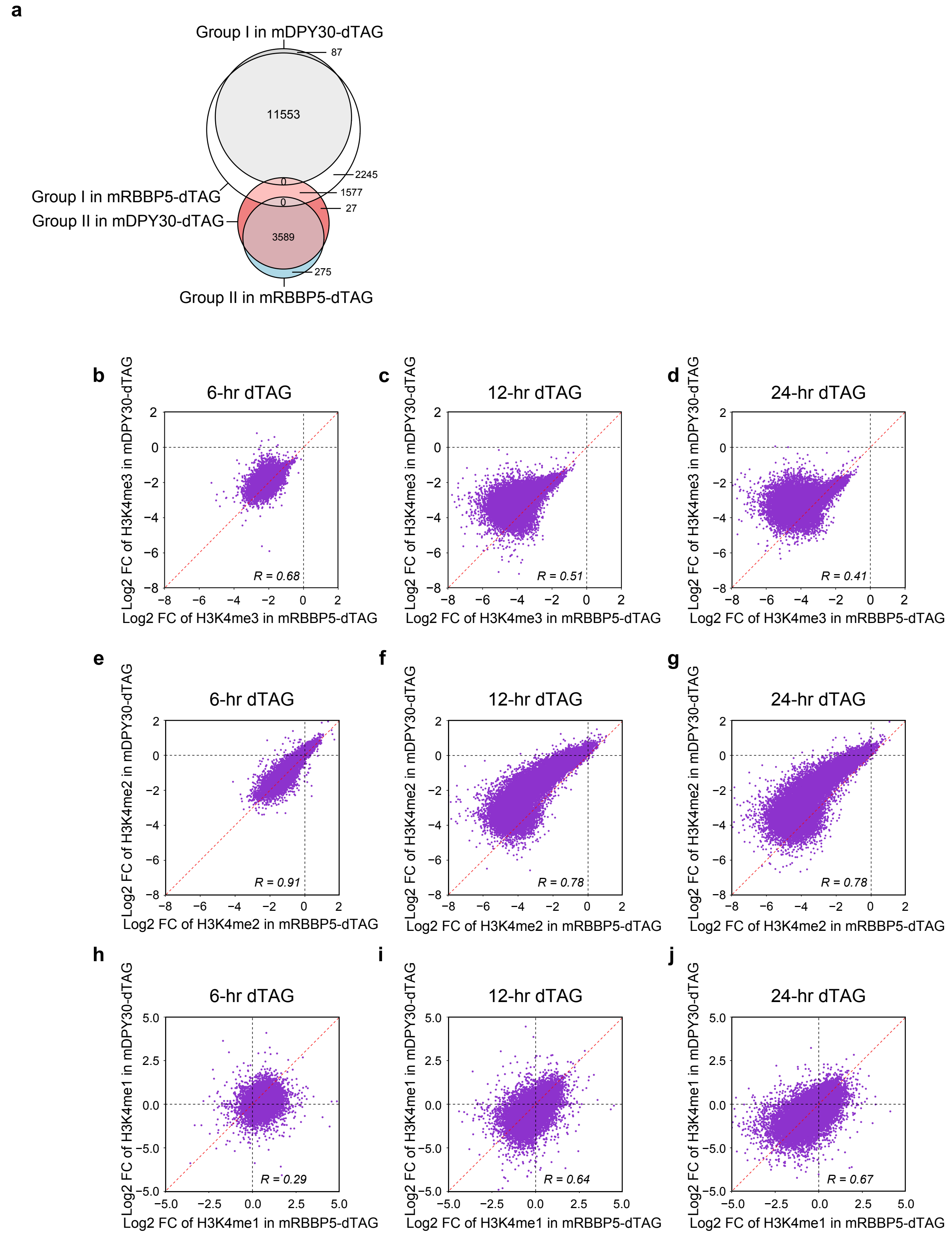

Supplementary information, Fig. S3: The reduction of H3K4me2 and H3K4me3 induced by RBBP5 depletion is more pronounced than by DPY30 depletion

a Venn diagram showing overlaps of Group I and Group II H3K4me3 peaks between mDPY30-dTAG mESCs and mRBBP5-dTAG mESCs. **b-d** Scatterplots depicting the correlations of the $\log_2$ fold change (FC) of H3K4me3 peaks of 6- (**b**), 12- (**c**), and 24-hour dTAG (**d**) versus DMSO treatment between mRBBP5-dTAG cells and mDPY30-dTAG cells. **e-g** Scatterplots depicting the correlations of the $\log_2$ fold change of H3K4me2 peaks of 6- (**e**), 12- (**f**), and 24-hour dTAG (**g**) versus DMSO treatment between mRBBP5-dTAG cells and mDPY30-dTAG cells. **h-j** Scatterplots depicting the correlations of the $\log_2$ fold change of H3K4me1 peaks of 6- (**h**), 12- (**i**), and 24-hour dTAG (**j**) versus DMSO treatment between mRBBP5-dTAG cells and mDPY30-dTAG cells.

Supplementary information, Fig. S4

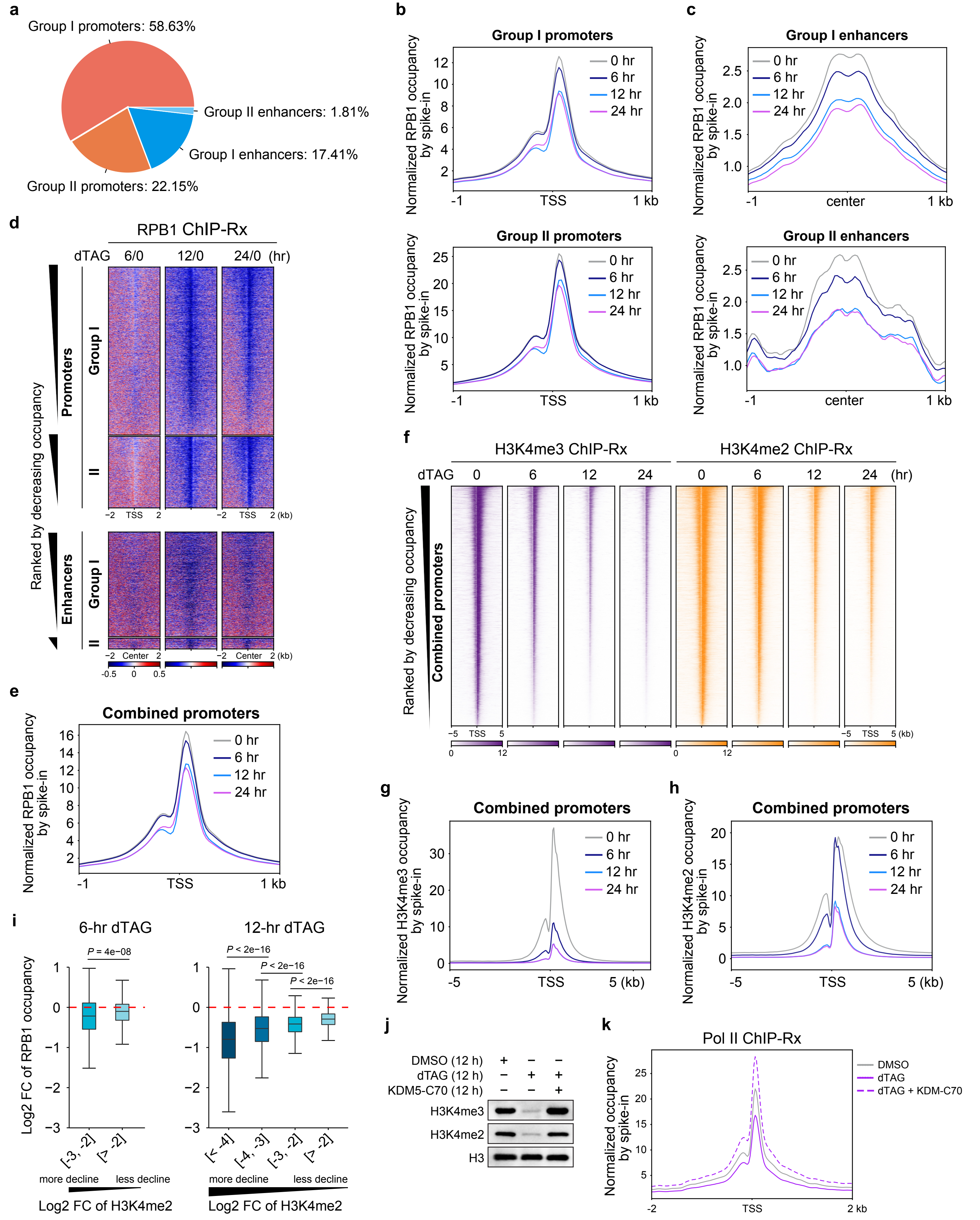

Supplementary information, Fig. S4: RBBP5 depletion and subsequent loss of H3K4me2 and H3K4me3 reduce Pol II levels at promoters

a Genomic distribution of Group I and Group II H3K4me3 peaks. **b** Metaplots showing the average Pol II occupancy at Group I (top) and Group II (bottom) promoters centered at the TSS of genes with H3K4me3 promoter peaks in mRBBP5-dTAG mESCs treated with dTAG for 0, 6, 12, and 24 hours. Pol II occupancy is represented by its largest subunit RPB1. **c** Metaplots showing the average Pol II occupancy at Group I (top) and Group II (bottom) enhancers centered at H3K4me3 peak centers in mRBBP5-dTAG mESCs treated with dTAG for 0, 6, 12, and 24 hours. **d** Heatmaps showing the log_2 fold change of Pol II signal (dTAG versus DMSO) at promoters and enhancers in mRBBP5-dTAG cells. **e** Metaplot showing the average RPB1 occupancy at combined promoters centered at the TSS of genes with H3K4me3 promoter peaks in mRBBP5-dTAG mESCs treated with dTAG for 0, 6, 12, and 24 hours. **f** Heatmaps showing the occupancies (RPM per bp) of H3K4me3 and H3K4me2 at combined promoters in mRBBP5-dTAG mESCs with indicated time-course of dTAG treatment. The peaks are ranked by decreasing H3K4me3 occupancy in the DMSO condition. **g, h** Metaplots showing the average H3K4me3 (**g**) and H3K4me2 (**h**) occupancies at combined promoters centered at the TSS of genes with H3K4me3 promoter peaks in mRBBP5-dTAG mESCs treated with dTAG for 0, 6, 12, and 24 hours. **i** Boxplots showing the correlation of the log_2 fold change of Pol II (dTAG versus DMSO) and H3K4me2 at promoters (dTAG versus DMSO, grouped based on the extent of declining H3K4me2 occupancy at promoters) in mRBBP5-dTAG mESCs. **j** Western blotting in mRBBP5-dTAG mESCs treated with dTAG and/or pan-KDM5 inhibitor KDM5-C70 for 12 hours. **k** Metaplot showing the occupancy of Pol II in mRBBP5-dTAG mESCs treated with dTAG and/or KDM5-C70 for 12 hours.

Supplementary information, Fig. S5

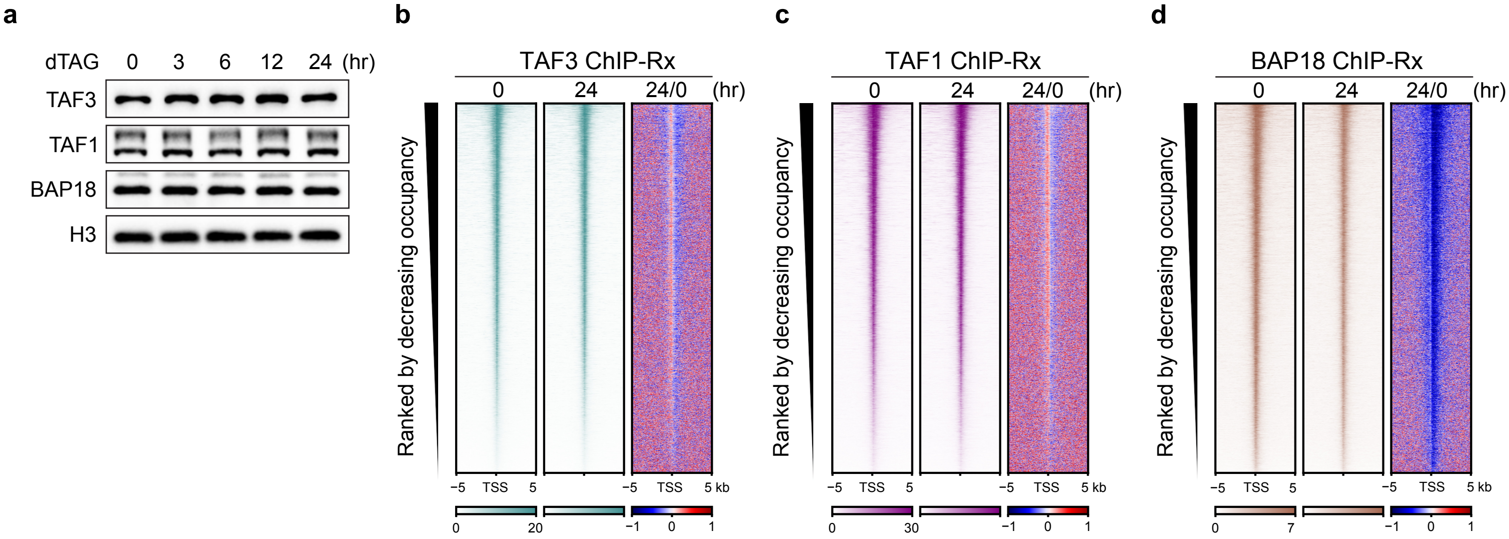

Supplementary information, Fig. S5: TFIID recruitment is not perturbed upon disruption of COMPASS and the subsequent loss of H3K4me2 and H3K4me3

a Western blotting of mRBBP5-dTAG mESCs with indicated time-course of dTAG treatment. **b-d** Heatmaps of TAF3 (**b**), TAF1 (**c**), and BAP18 (**d**) occupancies (RPM per bp and log_2 fold change) centered at the TSS of genes with H3K4me3 promoter peaks.

Supplementary information, Fig. S6

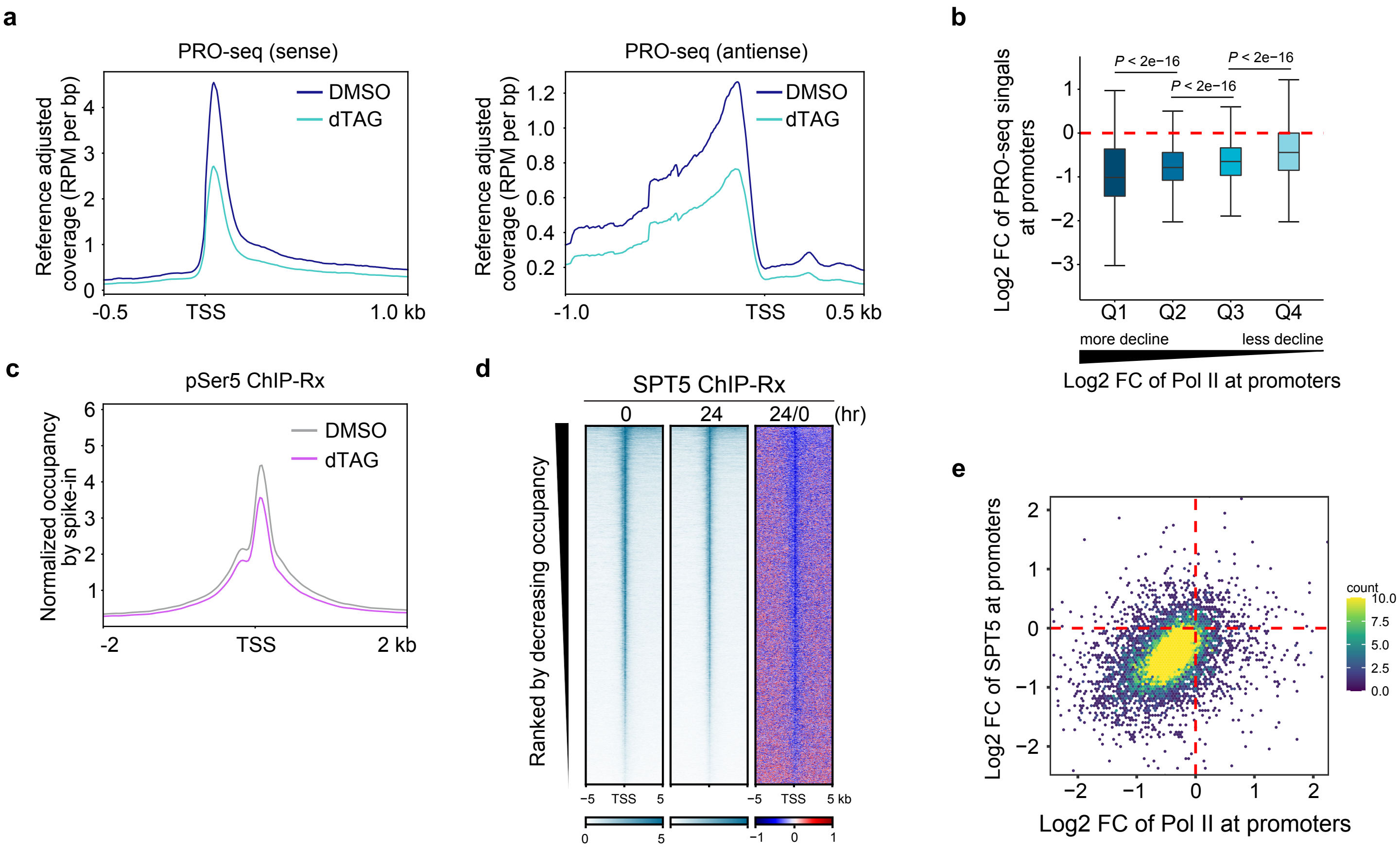

Supplementary information, Fig. S6: RBBP5 depletion and subsequent loss of H3K4me2 and H3K4me3 diminish the occupancy of paused Pol II

a Metaplot profiles of PRO-seq signal of sense and antisense transcription in DMSO- or dTAG-treated mRBBP5-dTAG mESCs. **b** Boxplot showing the correlation of the $\log_2$ fold change of PRO-seq signal (dTAG versus DMSO) and Pol II occupancy (dTAG versus DMSO) at promoters for four equal groups based on fold change of Pol II in mRBBP5-dTAG mESCs treated with DMSO or dTAG for 24 hours. **c** Metaplot showing the average occupancy of Pol II pSer5 in DMSO- or dTAG-treated mRBBP5-dTAG mESCs. **d** Heatmaps of SPT5 occupancy (RPM per bp and $\log_2$ fold change) centered at the TSS of genes with H3K4me3 promoter peaks ranked by decreased SPT5 occupancy in the DMSO condition. **e** Two-dimensional density plot comparing the $\log_2$ fold change of Pol II ChIP-Rx signal at promoters (x axis) and SPT5 signal at promoters (y axis) (dTAG versus DMSO) for genes with H3K4me3 promoter peaks.

Supplementary information, Fig. S7

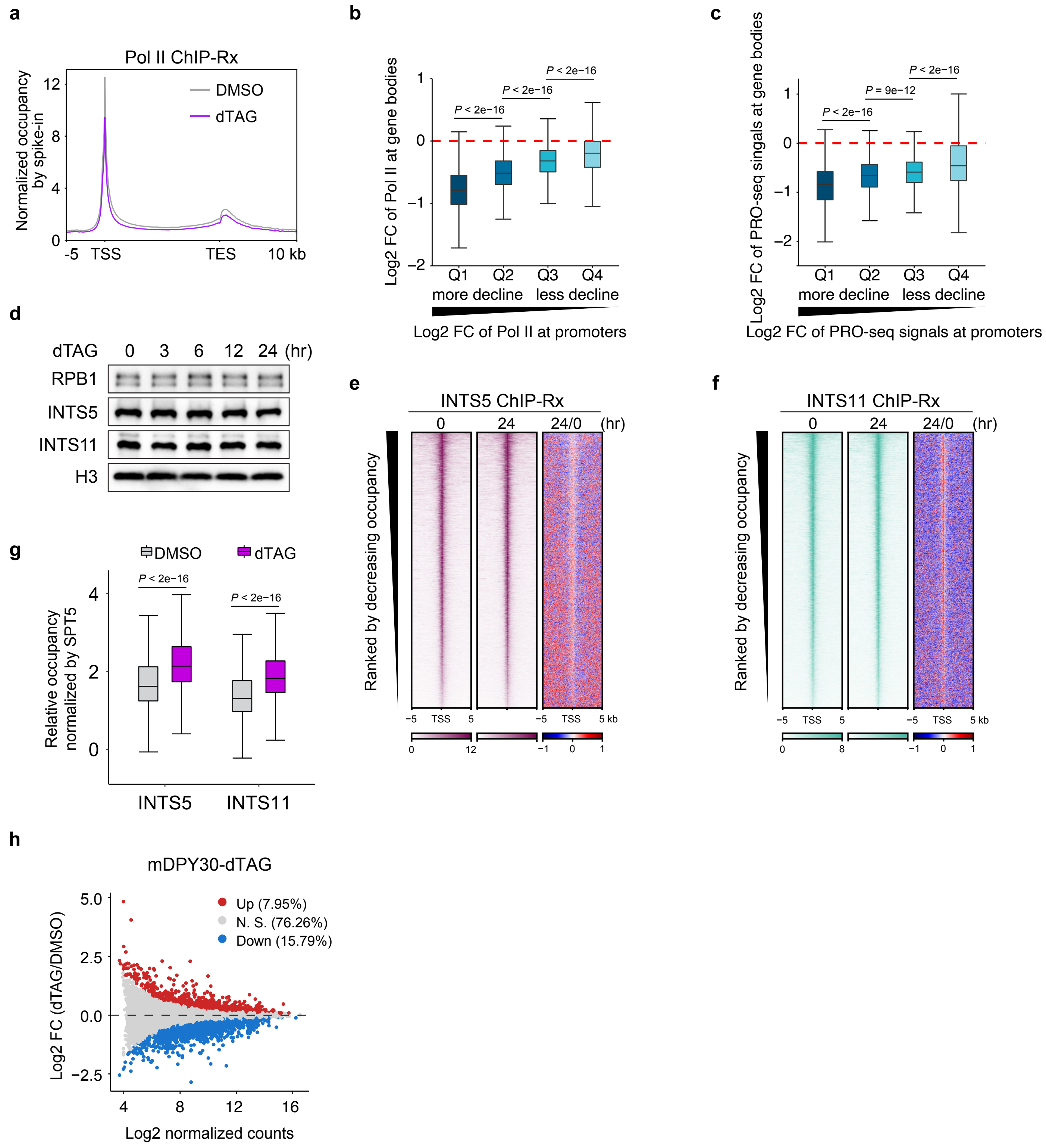

Supplementary information, Fig. S7: The loss of H3K4me2 and H3K4me3 compromise gene expression

a Metaplot showing the average Pol II occupancy across genes with H3K4me3 promoter peaks as measured by ChIP-Rx in mRBBP5-dTAG cells treated with DMSO or dTAG. **b** Boxplot showing the correlation of the log_2 fold change of Pol II at promoters and gene bodies (dTAG versus DMSO) for four equal groups based on the fold change of Pol II at promoters in mRBBP5-dTAG mESCs. **c** Boxplot showing the correlation of the log_2 fold change of PRO-seq signal at promoters and gene bodies (dTAG versus DMSO) for four equal groups based on the fold change of PRO-seq signal at promoters in mRBBP5-dTAG mESCs. **d** Western blotting of mRBBP5-dTAG mESCs with indicated time-course of dTAG treatment. **e, f** Heatmaps of INTS5 (**e**) and INTS11 (**f**) occupancies (RPM per bp and log_2 fold change) centered at the TSS of genes with H3K4me3 promoter peaks. **g** Boxplot showing the relative occupancies of INTS5 and INTS11 compared with SPT5 in mRBBP5-dTAG mESCs treated with DMSO or dTAG. **h** MA plot of spike-in normalized RNA-seq showing the gene expression changes by dTAG treatment for 24 hours in mDPY30-dTAG cells.

Materials and Methods

Culture of mESCs

MESCs were cultured in knockout DMEM (Gibco) with 15% fetal bovine serum (BVES500, Biovision), 1 mM L-glutamine (Gibco), 1× Sodium pyruvate (Gibco), 1× NEAA (Gibco), 0.11 mM β-mercaptoethanol (ES-007-E, Millipore), 1 μM PD0325901 (S1036, Selleck), 3 μM CHIR99021 (S1263, Selleck), 1000U/mL LIF (ESG1107, Millipore) and 1× penicillin/streptomycin (Gibco) at 37 °C and 5% CO2.

Antibodies and reagents

RBBP5 (CST, Cat.#13171S), DPY30 (Bethyl Labs, Cat.#A304-296A), H3K4me3 (Abclonal, Cat.#A2357), H3K4me2 (Abclonal, Cat.#A2356), H3K4me1 (Abclonal, Cat.#A2355), Histone H3 (Abclonal, Cat.#A17562), H3K27me3 (Abclonal, Cat.#), H3K9me3 (Active motif, Cat.#39161), H3K36me3 (CST, Cat.# 4909S), H3K79me3 (abcam, Cat.#ab2621), TAF1 (Bethyl Labs, Cat.#A303-505A), TAF3 (abcam, Cat.#ab188332), BAP18 (Bethyl Labs, Cat.#A304-207A), SPT5 (Proteintech, Cat.#16511-1-AP), Rpb1 NTD (CST, Cat.#16511-1-AP), Phospho-Rpb1 CTD (pSer5, Cat.#13523S) , INTS5 (Proteintech, Cat.#14069-1-AP), INTS11 (Bethyl Labs, Cat.# A301-274A), Anti-DYKDDDDK (Flag, Smart-lifesciences, Cat.#SLAB01), KDM5A (Active motif, Cat.#91211), KDM5B (abcam, Cat.#ab181089), KDM5C (Abclonal, Cat.#A15740), SETD1A (Bethyl Labs, Cat.#A300-289), SETD1B (CST, Cat.#44922).

dTAG-13 (Tocris, Cat.#6605), Triptolide (Cayman, Cat.#11973), KDM5-C70 (MCE, Cat.#HY-120400), Biotin-CTP (PerkinElmer, Cat. #NEL542001EA), Biotin-UTP (PerkinElmer, Cat. #NEL543001EA), 4-thiouridne (Cool Chemistry, Cat.#TU589689), MTSEA-biotin-XX (Biotium, Cat.#90064-1), Streptavidin Magnetic Beads (NEB, Cat.#S1420S), Puromycin(meilunbio, Cat.#MB2005), Cryopreservation Medium (Applied cell, Cat.#AC-1001041), VAHTS RNA Clean Beads (Vazyme, Cat.#N412-02), Fast RNA-seq Lib Prep Kit V2 (ABclonal, Cat.#RK20306), PAGE Gel Fast Preparation Kit (epizyme, Cat.#PG113).

Genome editing for endogenous knock-in dTAG cells

Endogenous knock-in of dTAG was conducted following previously described methods [1]. To generate endogenous knock-in mouse ES cells (mESCs), PITCh plasmids, sgRNA plasmids and donor plasmids

were transfected with Lipo3000 Reagents (Invitrogen) according to the manufacturer's instruction. 5×10^5 cells were seeded into 6-well plates one day in advance. 1 μg sgRNA-Cas9 plasmids, 1μg donor plasmids and 0.5 μg PITCh plasmids were mixed with P3000 and Lipo3000 in Opti-MEM. The mixture was incubated for 15 min at room temperature, and then added to the cell culture and incubated with cells overnight at 37 °C. Cells were trypsinized and seeded in 10 cm dishes with 1 μg/ml puromycin (Meilunbio) for 10-14 days. The surviving clones were picked, transferred to 96-well plates and expanded before genotyping by PCR. Protein degradation efficiencies of successful homogeneous clones were verified by dTAG-13 treatment followed by western blotting.

Chromatin immunoprecipitation sequencing (ChIP-seq) with reference exogenous genome (ChIP-Rx)

ChIP-Rx was performed as described previously [2]. 5×10^7 mESCs were crosslinked with 1% formaldehyde (Sigma) for 10 min at room temperature. The reaction was quenched by adding glycine to a final concentration of 125 mM and incubating for 5min. Cells were washed in cold PBS, resuspended in lysis buffer (50 mM HEPES pH7.4, 150 mM NaCl, 2 mM EDTA, 0.1% SDS, 0.1% sodium deoxycholate, 1 × protease inhibitor (Roche)). Fixed cells were sonicated (Qsonica) to obtain chromatin fragments between 200-700bp, and centrifuged to collect the soluble fraction. Human DLD-1 cells were used as a spike-in for normalization. Mixed lysates were then incubated overnight with antibodies and magnetic Protein A/G beads for 2 hours. The beads were washed 3 times with High Salt Wash buffer (20 mM HEPES pH7.4, 500 mM NaCl, 1 mM EDTA, 1.0% NP-40, 0.25% sodium deoxycholate), twice with Low Salt Wash buffer (20 mM HEPES pH 7.4, 150 mM NaCl, 1 mM EDTA, 0.5% NP-40, 0.1% sodium deoxycholate), and once with TE buffer containing 50 mM NaCl. Beads were eluded with Elution buffer (50 mM Tris-HCl pH 8.0, 10 mM EDTA, 1.0% SDS). The eluted samples were digested with Proteinase K and purified by Phenol/Chloroform/Isoamyl Alcohol extraction. The sequencing libraries were prepared with VAHTS Universal Plus DNA Library Prep Kit for Illumina (Vazymes). Libraries were sequenced using an Illumina HiSeq X Ten or NovaSeq 6000 platform (Annoroad Gene Technology, Beijing, China).

Transient transcriptome sequencing (TT-seq)

TT-seq was carried out as described previously [3]. mESCs were grown in 15 cm dishes, and the RNA was labeled with 4-thiouridine (4sU) (Cool Chemistry) in vivo for 15 min. TRIzol (Invitrogen) was added to stop the reaction and total RNA was extracted according to instructions. As a control, 4sU-labeled DLD-1 RNA was mixed as the spike-in. The mix was fragmented with 0.2 M NaOH for 14min and Tris-HCl (pH 6.8) was added to stop the fragmentation. Biotinylation of labeled RNA was carried out in 150 μL of biotinylation mix (100 μg fragmented total RNA, 10 mM HEPES pH 7.5, 1 mM EDTA, 0.167 mg/mL MTSEA-biotin) for 30 min and purified with Streptavidin Magnetic Beads (NEB). The biotinylated RNA was eluted with 100 mM DTT and purified with RNA Clean beads (Vazymes). The RNA libraries were constructed from the 200 μg purified RNA sample using Stranded mRNA-seq Lib Prep Module for Illumina (ABclonal, RK20349) following the guidelines of the manufacturers and sequenced with a NovaSeq 6000 platform (Annoroad Gene Technology, Beijing, China).

Precision run-on sequencing (PRO-seq)

PRO-seq was performed according to the previously published protocol with minor modifications [3,4]. mESCs were rinsed twice with 5 ml of cold 1 × PBS and scraped with 5ml permeabilization buffer (10 mM Tris-HCl pH 8.0, 5% glycerol, 250 mM sucrose, 10 mM KCl, 5 mM $MgCl_2$, 1 mM EGTA, 0.5 mM DTT, 0.1% Igepal, 0.05% Tween-20, protease inhibitors cocktail (Roche), 4 U/mL RNase inhibitor (SUPERaseIN)). The resuspended cells were incubated for up to 5min on ice, then transferred for centrifugation and washing in ice-cold wash buffer (10 mM Tris-HCl pH 8.0, 10 mM KCl, 5% glycerol, 5 mM MgCl2, 0.5 mM DTT, 4 U/mL RNase inhibitor). Permeabilized cells were resuspended in freezing buffer (50 mM Tris-HCl pH 8.0, 40% glycerol, 5 mM MgCl2, 1 mM EDTA, 0.5 mM DTT, 4 U/mL RNase inhibitor) and immediately frozen in liquid nitrogen. The permeabilized cells were stored at -80 °C until usage.

Permeabilized cells were mixed with spike-in DLD-1 cells. Nuclear run-on reactions were performed with 2 × nuclear run-on reaction mixture (10 mM Tris-HCl pH 8.0, 300 mM KCl, 1% Sarkosyl, 5 mM MgCl2, 1 mM DTT, 40 μM Biotin-11-A/C/G/UTP (Perkin-Elmer), 0.8 U/mL RNase inhibitor) and incubated for

5min at 37°C. Nascent RNA was extracted by Trizol LS Reagent (Invitrogen). RNA fragmentation was performed with 0.25N NaOH on ice for 10 min and neutralized by adding 1M Tris-HCl pH6.8, followed by passing through a calibrated RNase-free P-30 column (Bio-Rad). After 3' RNA adaptor ligation, RNA was purified by Streptavidin Magnetic Beads (NEB) in Binding buffer (10 mM Tris-HCl pH 7.4, 300 mM NaCl, 0.1% Triton X-100, 1 mM EDTA). The beads were washed once with High Salt Wash buffer (50 mM Tris-HCl pH 7.4, 2 M NaCl, 0.5% Triton X-100,1 mM EDTA) and once with Low Salt Wash buffer (5 mM Tris-HCl pH 7.4, 0.1% Triton X-100, 1 mM EDTA). On-Bead RNA 5' hydroxyl repair was then performed with PNK mix (1 × PNK Buffer, 1 mM ATP, 10 U PNK) at 37 °C for 30 min. The RNA 5' decapping was performed with RppH mix (1 × ThermoPol Buffer, 5 U RppH) at 37 °C for 1 h. On-Bead 5' RNA adaptor ligation was performed by the ligation mix (1 × T4 RNA ligase buffer, 1 mM ATP, 15% PEG 8000, 10 U T4 RNA Ligase I) at 25 °C for 1 hour, followed by TRIzol elution of the adaptor ligated RNA. The RNA was reverse transcribed by the Maxima H Minus RT enzyme (Invitrogen). Full-scale libraries were amplified with Q5 enzyme mix (NEB). Libraries were sequenced with a NovaSeq 6000 platform (Annoroad Gene Technology, Beijing, China).

Quantification and statistical analysis

Identification of promoter and enhancer

Genome annotation and reference genome sequences were downloaded from the UCSC Genome Browser. The longest transcript isoform of each alternatively spliced RefSeq protein-coding gene [5] was selected as the representative transcript. The chosen transcripts and their transcription start sites (TSS) were then used for further analyses. For ChIP-Rx data, we defined the promoter region as TSS – 1000 bp to TSS + 1000 bp, the genebody region as TSS + 3000 bp to TES, and the intergenic enhancer region as from 2kb upstream and downstream of the protein-coding genes. For PRO-seq data, we defined the promoter region as TSS – 10 bp to TSS + 300 bp and the genebody region as TSS + 300 bp to TES.

ChIP-Rx data analysis

The raw ChIP-Rx reads were trimmed by Trim Galore v0.6.6 (https://www.bioinformatics.babraham.ac.uk/projects/trim_galore/) and aligned to the human hg19 and mouse mm10 assemblies using Bowtie v2.3.5.1 with default parameters [6]. Low mapping quality reads (MAPQ < 30) and PCR duplicates were removed using SAMtools v1.9 [7] and Picard v2.23.3 (https://broadinstitute.github.io/picard/). We then collected the spike-in read number for each of the ChIP-Rx samples with SAMtools v1.9 [7] and generated the normalization factor as 1e6/spike-in_count. Normalized bigwig files were generated by deeptools v3.5.0 [8]. The ENCODE blacklist regions were removed using bedtools v2.29.2 [9]. Peak calling was performed by macs2 v2.2.6 with a q-value threshold of 0.05 [10]. DiffBind R package v2.16.2 [11] was used to identify differential binding peaks.

PRO-seq data analysis

The paired PRO-seq reads were trimmed by Trim Galore v0.6.6 (https://www.bioinformatics.babraham.ac.uk/projects/trim_galore/) with read length >15 bp. After removal of molecular barcode (UMI) with fastp v0.21.0 [12], ribosomal RNA reads were discarded using Bowtie v2.3.5.1 with ‘‘--un-conc-gz’’ [6]. Then the remaining reads were aligned to the hg19 or mm10 genome using Bowtie v2.3.5.1 with ‘‘--local --sensitive-local’’ [6]. The mapped data were next deduplicated with UMI-tools to remove PCR duplicates based on the UMI sequences [13]. We generated the normalization factor as described in ChIP-Rx and built strand-specific coverage tracks with deeptools v3.5.0 [8].

RNA-seq data analysis

Raw reads were trimmed as described for ChIP-Rx, followed by mapping to the hg19 or mm10 genome using STAR v2.7.5c with parameter ‘‘--outSAMtype BAM SortedByCoordinate --twopassMode Basic --outFilterMismatchNmax 3’’ [14]. Low mapping quality reads and PCR duplicates were removed using SAMtools v1.9 [7] and Picard v2.23.3 (https://broadinstitute.github.io/picard/). Calculation of the normalization factor was performed in the same way as described in ChIP-Rx. Strand-specific normalized bigwigs were generated by deeptools v3.5.0 [8]. To identify differentially expressed genes, we collected the

counts per gene by the featureCounts tool from Rsubread R package v2.0.1 [15]. Differential expression analysis was then performed using DESeq2 R package v1.26.0 [16].

References

1 Nabet, B. *et al.* The dTAG system for immediate and target-specific protein degradation. *Nat Chem Biol* **14**, 431-441, doi:10.1038/s41589-018-0021-8 (2018).

2 Orlando, D. A. *et al.* Quantitative ChIP-Seq normalization reveals global modulation of the epigenome. *Cell Rep* **9**, 1163-1170, doi:10.1016/j.celrep.2014.10.018 (2014).

3 Schwalb, B. *et al.* TT-seq maps the human transient transcriptome. *Science* **352**, 1225-1228, doi:10.1126/science.aad9841 (2016).

4 Judd, J. *et al.* A rapid, sensitive, scalable method for Precision Run-On sequencing (PRO-seq). *bioRxiv*, 2020.2005.2018.102277, doi:10.1101/2020.05.18.102277 (2020).

5 O'Leary, N. A. *et al.* Reference sequence (RefSeq) database at NCBI: current status, taxonomic expansion, and functional annotation. *Nucleic Acids Res* **44**, D733-745, doi:10.1093/nar/gkv1189 (2016).

6 Langmead, B. & Salzberg, S. L. Fast gapped-read alignment with Bowtie 2. *Nat Methods* **9**, 357-359, doi:10.1038/nmeth.1923 (2012).

7 Li, H. *et al.* The Sequence Alignment/Map format and SAMtools. *Bioinformatics* **25**, 2078-2079, doi:10.1093/bioinformatics/btp352 (2009).

8 Ramirez, F. *et al.* deepTools2: a next generation web server for deep-sequencing data analysis. *Nucleic Acids Res* **44**, W160-165, doi:10.1093/nar/gkw257 (2016).

9 Quinlan, A. R. & Hall, I. M. BEDTools: a flexible suite of utilities for comparing genomic features. *Bioinformatics* **26**, 841-842, doi:10.1093/bioinformatics/btq033 (2010).

10 Zhang, Y. *et al.* Model-based analysis of ChIP-Seq (MACS). *Genome Biol* **9**, R137, doi:10.1186/gb-2008-9-9-r137 (2008).

11 Ross-Innes, C. S. *et al.* Differential oestrogen receptor binding is associated with clinical outcome in breast cancer. *Nature* **481**, 389-U177, doi:10.1038/nature10730 (2012).

12 Chen, S., Zhou, Y., Chen, Y. & Gu, J. fastp: an ultra-fast all-in-one FASTQ preprocessor. *Bioinformatics* **34**, i884-i890, doi:10.1093/bioinformatics/bty560 (2018).

13 Smith, T., Heger, A. & Sudbery, I. UMI-tools: modeling sequencing errors in Unique Molecular Identifiers to improve quantification accuracy. *Genome Research* **27**, 491-499, doi:10.1101/gr.209601.116 (2017).

14 Dobin, A. *et al.* STAR: ultrafast universal RNA-seq aligner. *Bioinformatics* **29**, 15-21, doi:10.1093/bioinformatics/bts635 (2013).

15 Liao, Y., Smyth, G. K. & Shi, W. The R package Rsubread is easier, faster, cheaper and better for alignment and quantification of RNA sequencing reads. *Nucleic Acids Res* **47**, e47, doi:10.1093/nar/gkz114 (2019).

16 Love, M. I., Huber, W. & Anders, S. Moderated estimation of fold change and dispersion for RNA-seq data with DESeq2. *Genome Biol* **15**, 550, doi:10.1186/s13059-014-0550-8 (2014).
